# Novel TrueVue series of 3D echocardiography: Revealing the pathological morphology of congenital heart disease

**DOI:** 10.3389/fphys.2022.1000007

**Published:** 2022-09-06

**Authors:** Feifei Sun, Aijiao Sun, Yixin Chen, Yangjie Xiao, Xintong Zhang, Wei Qiao, Xueying Tan, Yanxiao Liang, Dongyu Li, Shu Yang, Weidong Ren

**Affiliations:** ^1^ Department of Ultrasound, Shengjing Hospital of China Medical University, Shenyang, China; ^2^ Department of Cardiac Surgery, Shengjing Hospital of China Medical University, Shenyang, China; ^3^ Department of Ultrasound, Philips Medical Technology, Shenyang, China

**Keywords:** heart defects, congenital abnormalities, echocardiography, real-time three-dimensional, ultrasonic imaging

## Abstract

**Aims:** This study explored the advantages and limitations of novel series of three-dimensional (3D) echocardiographic techniques and summarized their application methods for congenital heart diseases (CHDs).

**Method and result:** Two-dimensional (2D), traditional 3D echocardiography, and TrueVue plus light and/or Glass novel 3D technologies were performed on 62 patients with CHD, and a clinical survey was designed to judge whether the novel 3D images were more helpful for understanding the cardiac condition and guide treatment than traditional 3D images. TrueVue increased the visual resolution and simulated the true texture of cardiac tissue, significantly improving the display ability of abnormal anatomical structures in CHDs. TrueVue Glass displayed the blood channel and the internal structure of cardiac cavity more intuitively, indicating a new observation aspect not shown by conventional echocardiography. The clinical survey results showed that the new 3D imaging methods effectively increased the diagnostic confidence of echocardiographers, enabled surgeons to better understand the details of lesions, promoted efficient communication, and improved the confidence of both doctors and patients in treatment.

**Conclusion:** The combined application of TrueVue, TrueVue Light, and TrueVue Glass more closely simulated real anatomical features, showed more comprehensive and subtle blood flow in the lumen, not only increased the visual effect but also provided more useful diagnostic information, improved the accuracy of evaluation and treatment of CHD when compared to traditional imaging techniques, indicating that this combined application has significant clinical value.

## Introduction

Congenital heart disease (CHD) may be caused by developmental disorders during the cardiac embryonic period or unclosed channels after birth and is an important cause of death for children and adults (Meras et al., 2021). Real-time three-dimensional echocardiography (3DE) can visually and stereoscopically display some congenital malformation, providing supplementary information for the diagnostic evaluation of various CHDs. However, traditional 3DE is affected by many factors, such as limited image quality and a monotonous imaging mode (Cossor et al., 2015; Simpson et al., 2017). It has been considered that it cannot be used as an independent diagnostic tool, and reliance on 2D dynamic scanning remains prevalent (Fabricius et al., 2004; van den Bosch et al., 2006; Simpson et al., 2017). Recently, the advent of the TrueVue, TrueVue Light, and TrueVue Glass series of novel rendering technologies (from Philips company) provides more comprehensive, advanced 3D diagnostic evidence for evaluating abnormal cardiac anatomical structures (Genovese et al., 2019; Kern et al., 2019; Vainrib et al., 2019; Vairo et al., 2019). Especially for the artificial intelligence (AI) technology-assisted TrueVue Glass imaging mode, which released globally in the second half of 2020, there are few applied studies of these technologies, and they are mainly limited to transesophageal echocardiography (TEE) (Karagodin et al., 2020); to our knowledge, there has been no systematic exploration of their application for CHD. This study is the first to jointly apply the TrueVue, TrueVue Light, and TrueVue Glass series of advanced 3DE to diagnose and evaluate CHD and explore their application methods and practical value. In addition, a questionnaire was employed to evaluate the merit of these novel tools to echocardiographers, cardiac surgeons, and patients.

## Materials and methods

### Patients’ data collection

This study included 62 patients, who underwent echocardiographic evaluation in our hospital for cardiac-related clinical symptoms or signs and were diagnosed with CHD from August 2020 to May 2021, including 30 males and 32 females, with a mean age of 15.56 ± 14.71 years (range: 3 days to 56 years). This study does not establish strict exclusion criteria, even if the quality of the 2D image is not very satisfactory. For the original 2D image quality is not ideal, we also try to compare the imaging effect and the improvement of the diagnostic value after the conversion to the new 3D imaging mode, in order to objectively evaluate the actual value of these new imaging technologies. Therefore, TTE was performed in 41 cases and TEE was performed in 21 cases. TEE examiners excluded contraindications. All patients or their legal guardians signed an informed consent form. This study was approved by the Ethics Committee of China Medical University. The study protocol conforms to the ethical guidelines of the 1975 Declaration of Helsinki as reflected in *a priori* approval by the institution’s human research committee.

### Novel three-dimensional echocardiography image acquisition

Imaging was obtained via Philips EPIQ system, CVx equipment (Philips, Andover, MA), with the appropriate 3D probes, including X5-1 or X7-2 for transthoracic echocardiography (TTE) or X8-2t for TEE. Each imaging mode was set to store continuous four cardiac cycles. After the 2D image was satisfactorily obtained, traditional 3D acquisition modes were initiated, such as Live 3D, Full Volume, or 3D Zoom to obtain real-time 3D images (Sun et al., 2017), and six-sided cutting (Box Crop), free surface cutting (Plane Crop), frontal cutting (Face Crop), quick cutting (Quick Crop), or intelligent cutting (iCrop) were used to highlight the target lesion. TrueVue was then initiated to obtain a high-definition photorealistic stereoscopic image. After clicking “Touch”, the 3D image appears not only on the monitor, but also on the operation panel at the bottom of the monitor; then, the user can click directly on the operation panel to add a light source at a specific location, move the position of the light source, adjust the depth, and/or touch to adjust the image angle and size to obtain a more vivid image. Engaging the Glass mode provides a transparent image of the heart. These 3D image modes can be optimized for grayscale, brightness, smoothness, contrast, or transparency according to the target structure characteristics.

### Clinical survey

We randomly selected 10 sonographers (the average age is 35.6 ± 10.73 years) as the echocardiographer group, eight doctors (40 ± 8.9 years) who specialize in cardiac intervention or thoracotomy surgery as the cardiac surgeon group (no limitation on working years, including doctors who have worked for 1–25 years, the overall average age is 37.6 ± 9.7 years), six cardiologists specializing in CHD interventional techniques (unlimited years of experience, including worked for 1–30 years, mean age 32.5 ± 11.3 years), and 20 patients with CHD (or a parent of a child under 14 years) who underwent the novel 3D echocardiographic examinations to fill out a short questionnaire evaluating their experience, they were all provided score [using Likert score system (Harake et al., 2020)] the elements after viewing/manipulate the new series of 3D images/videos by comparing with the traditional 3D for 30 cases randomly selected from the database (Table 1). None of the participants had prior experience with the TrueVue series of technologies. The cases selected for evaluation should be avoided as far as possible to be biased towards those images that will be improved by new rendering display. Instead, we chose a collection of various congenital heart diseases that we encountered randomly during a certain period of clinical work.

**TABLE 1:** Scoring results of each group for three-dimensional echocardiography.

Compared with 2DE, the imaging effects of the 3DE showed (*N* = 62^a^)	Echocardiographers (*n* = 10)			Surgeons (*n* = 8)			Cardiologists (*n* = 6)			Patients or their parents (*n* = 20)		
	Likert score*			Likert score*			Likert score*			Likert score*		
	Conventional	New series	*p*	Conventional	New series	*p*	Conventional	New series	*p*	Conventional	New series	*p*
	3DE	of 3DE	value	3DE	of 3DE	value	3DE	of 3DE	value	3DE	of 3DE	value
Display the details of the lesion is more vivid and clearer	3 (2, 3)	4 (3, 5)	**<0.0001**	3 (1, 3)	5 (4, 5)	**<0.0001**	3 (2, 3)	5 (4, 5)	**<0.0001**	NC	NC	NC
The boundaries of the septal defect are clearly shown, and the 3D images are very similar to those seen intraoperatively^b^	3 (3, 4)	5 (4, 5)	**0.0001**	3 (2, 3)	5 (4, 5)	**<0.0001**	2 (2, 3)	5 (4, 5)	**<0.0001**	NC	NC	NC
After 3D combined with color Doppler, the entire length and stereoscopic extent of the abnormal shunt passing through the defect is displayed more clearly and accurately^b^	2 (2, 3)	5 (4, 5)	**<0.0001**	2 (1, 3)	5 (4, 5)	**<0.0001**	2 (1, 3)	4 (4, 5)	**<0.0001**	NC	NC	NC
The determination of the location, number and extent of congenital leaflet clefts is more rapid and effective^c^	2 (2, 3)	5 (4, 5)	**<0.0001**	2 (1, 3)	5 (4, 5)	**0.001**	2 (1, 3)	4 (4, 5)	**<0.0001**	NC	NC	NC
Can provide valuable stereoscopic images of the PV, including leaflet number and activity status^d^	2 (1, 2)	4 (3, 5)	**0.003**	2 (1, 2)	4 (3, 5)	**<0.0001**	2 (1, 2)	4 (3, 5)	**0.001**	NC	NC	NC
The entire pathway of blood flow in the lumen of an abnormally located vessel can be seen more clearly	2 (1.2, 2)	5 (3.5, 5)	**<0.0001**	2 (1, 3)	4 (4, 5)	**<0.0001**	2 (1.3, 2)	4 (3, 5)	**0.002**	NC	NC	NC
The number and connection characteristics of the abnormal atrioventricular valves can be clearly demonstrated^e^	2 (2, 3)	4 (4, 5)	**0.004**	3 (2, 3)	5 (4, 5)	**<0.0001**	3 (2, 3)	4 (4, 5)	**0.02**	NC	NC	NC
More helpful in the perception of the level and depth of the lesion	3 (2, 3)	5 (4, 5)	**<0.0001**	3 (2, 3)	4 (3, 5)	**<0.0001**	2 (1, 2)	4 (3, 5)	**0.004**	NC	NC	NC
A quicker diagnosis was made with the aid of 3D imaging	4 (3, 4)	5 (4, 5)	**0.04**	4 (3, 4)	5 (4, 5)	0.13	3 (2, 4)	5 (4, 5)	**0.002**	NC	NC	NC
The pathological pattern of the abnormal anatomy is well understood and grasped prior to surgery	3 (2, 5)	5 (4, 5)	**<0.0001**	3 (3, 4)	5 (4, 5)	**0.04**	4 (3, 4)	5 (4, 5)	0.27	2 (2, 3)	4 (4, 5)	**<0.0001**
Communication efficiency is enhanced by showing the patient or guardians the 3D image of the lesion	4 (3, 4)	5 (4, 5)	**0.04**	3 (3, 4)	5 (4, 5)	**<0.0001**	2 (1, 3)	4 (3, 5)	**<0.0001**	3 (2, 3)	4 (3, 5)	**0.002**
Increased my confidence in diagnosis and/or the success of the operation	3 (3, 4)	5 (4, 5)	**<0.0001**	2 (2, 3)	5 (4, 5)	**0.001**	2 (2, 3)	4 (4, 5)	**<0.0001**	2 (2, 3)	4 (3.5, 5)	**<0.0001**
Overall Score	2 (2, 3)	5 (3, 5)	**<0.0001**	3 (2, 3)	5 (4, 5)	**<0.0001**	2 (1, 3)	4 (4, 5)	**<0.0001**	2 (2, 3)	4 (4, 5)	**<0.0001**

a Each observer randomly evaluated the images and videos from the database for each case, during the evaluation process, the images were transformed and post-processed with the assistance of “doctors experienced in new 3DE” to achieve near-ideal image quality.

b This question applies only to cases of atrial septal defects and ventricular septal defects.

c This question applies only to patients with mitral valve cleft.

d This question applies only to patients with pulmonary valve stenosis.

e This question is only applicable in cases of complete atrioventricular septal defects.

*A 5-point scoring system, 1 indicating strongly disagree and 5 indicating strongly agree. Scores are expressed as median (IQR). *p* less than 0.05 was considered statistically significant. 2DE, two-dimensional echocardiography; 3D, three-dimensional; NC, no collection; PV, pulmonary valve.

Bolded values in the *p* column represent statistically significant results.

### Statistics

Using SPSS 25.0 software (IBM Inc., Armonk, New York, United States), categorical data are expressed as number of cases (percentage), for analysis of the Likert responses to the question design, data were tested for normality using the Kolmogorov–Smirnov test. Results are reported as mean ± standard deviation and median [first interquartile range (IQR), third interquartile range] for normally distributed and non-normally distributed data, respectively. Continuous normally-distributed data were compared using Student’s *t*-test. Non-Gaussian data were compared using the Mann-Whitney test. Comparisons among the participant groups were performed using analysis of variance, the Kruskal-Wallis test, or the Chi square test as appropriate, and *p* < 0.05 was considered statistically significant.

## Results

### Patient demographics

The advanced novel 3D echocardiography classified the 62 patients as follows: 18 (29.0%) with ventricular septal defect (VSD); 17 (27.4%) with atrial septal defect (ASD); 9 (14.5%) with patent ductus arteriosus (PDA); 5 (8.1%) with aortic valve malformation; 4 (6.5%) with mitral valve cleft; 3 (4.8%) with pulmonary artery stenosis; 2 (3.2%) with complete atrioventricular septal defect (CAVSD); 2 (3.2%) with double orifice mitral valve (DOMV); and one case (1.6%) each of pulmonary artery sling, and ventricular diverticulum. Of the total patients, 55 (88.7%) underwent cardiac surgery, including conventional thoracotomy or interventional cardiac procedures, wherein the results of the ultrasound diagnosis were confirmed.

### Novel three-dimensional echocardiographic analysis

**FIGURE 1:**
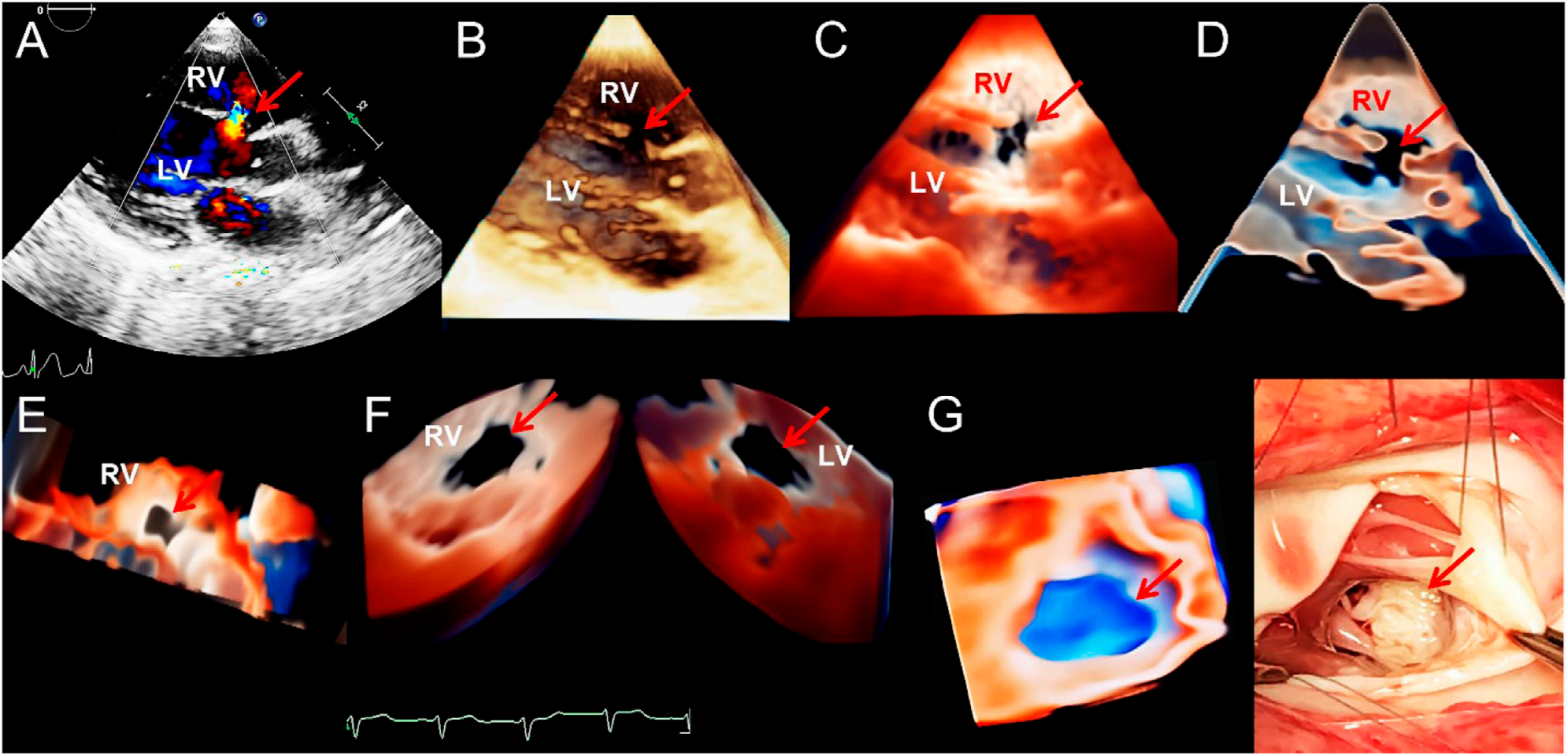
Transthoracic echocardiogram showing ventricular septal defect. **(A–D)**. On long axis section of the left ventricle, the 2D, traditional 3D, TrueVue Light, and TrueVue Glass images indicate the defects of the peri-membranous part of the interventricular septum (5 mm × 10 mm, arrows), respectively. **(E)**. In the same patient as A-D, looking down the defect from the right ventricle directly through a single view. **(F)**. In another patient with a large (22 mm × 14 mm) peri-membranous ventricularseptal defect, the “Dual Volume” imaging mode in TrueVue was used to observe the defects (arrows) directly from the right and left ventricles simultaneously. **(G)**. It is the same patient as in Figure **(F)**, showing the three-dimensional shape of the defect displayed by using TrueVue Glass to simulate the “surgical view”(left, arrow), and the comparison of the doctor’s field of vision during cardiac surgery (right, arrow). In surgery, after lifting the tricuspid valve, the mitral valve can be seen through the septal defect. 2D, two-dimensional; 3D, three-dimensional; LV, left ventricle; RV, right ventricle.

**FIGURE 2:**
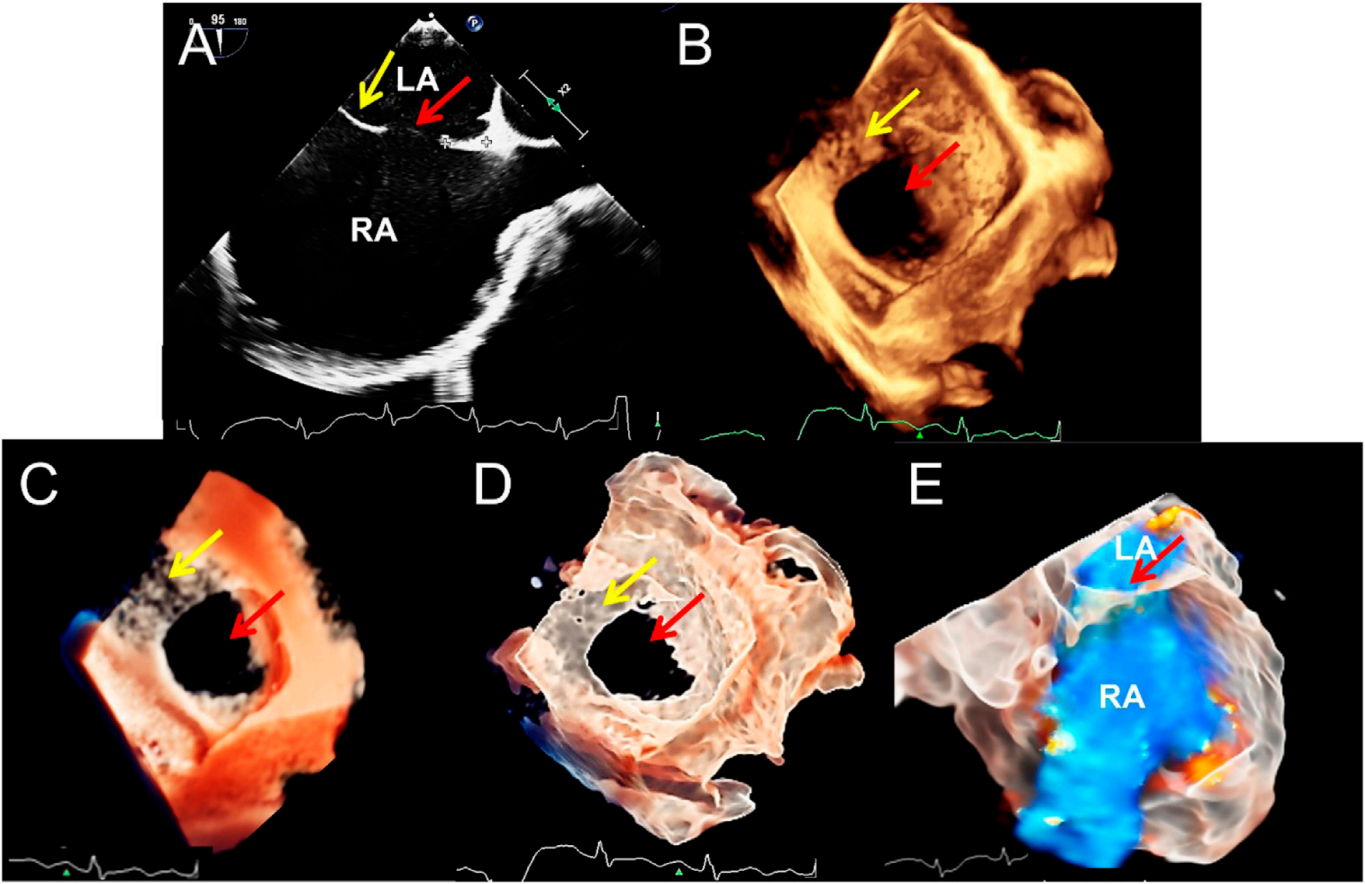
Transesophageal echocardiography of double atrial-superior and inferior vena cava section to diagnose atrial septal defect. **(A–D)**. The 2D, traditional 3D, TrueVue Light, and TrueVue Glass show the secondary type of atrial septal defect (25 mm × 23 mm, red arrows) and the upper soft, thin rim (yellow arrows), respectively. Figure **(B–D)** is a direct view of the atrial septum from the left atrium perspective. **(E)**. TrueVue Glass shows the spatial path of the atrial shunt from left to right. 2D, two-dimensional; 3D, three-dimensional; LA, left atrium; RA, right atrium.

**FIGURE 3:**
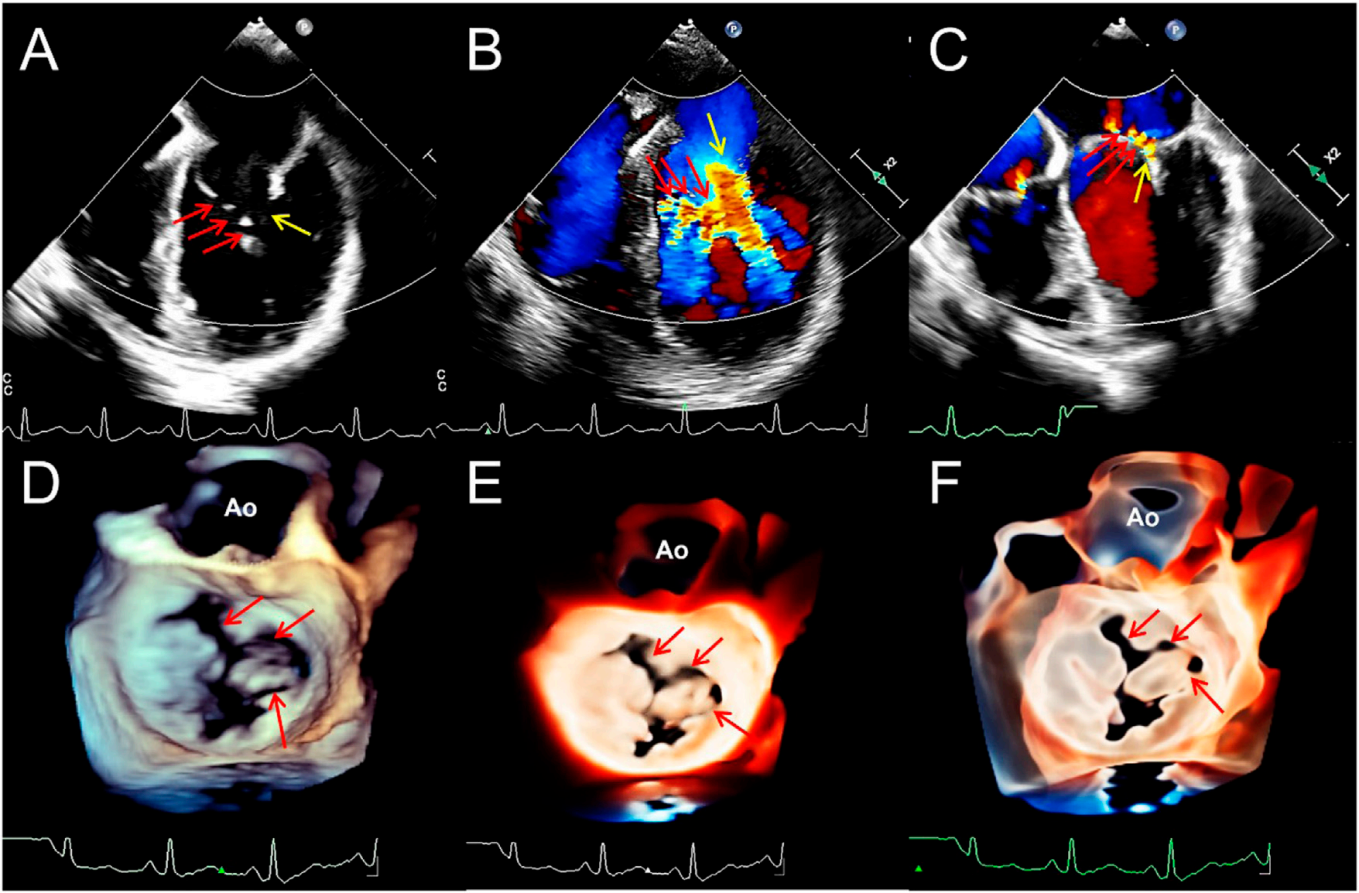
Transesophageal echocardiography reveals multiple mitral valve clefts. **(A)**. 2D echocardiography four-chamber view showing multiple loss of echo in the anterior leaflet of mitral valve (MV) (the sizes of the clefts from A1 to A3 area are 3.1, 4.6, and 3.4 mm respectively, red arrows). The yellow arrow points to the junction between the anterior and posterior leaflets. **(B,C)**. Blood flows into the left ventricle during diastole (red arrows) and regurgitation jets into left atrium during systole (red arrows) from the clefts. Yellow arrows represent the blood flow signals of the normal MV orifices. (**D–F)**. From a surgical perspective, traditional 3D, TrueVue Light, and TrueVue Glass show three irregular clefts (the range of the clefts from A1 to A3 area are 2.5–5.4, 2.5–4.3, and 2.0–3.3 mm, respectively) from the perspective of the left atrium, and all are complete cleft to the annulus. 2D, two-dimensional; 3D, three-dimensional; AO, Aorta; A1, A1 scallop of MV; A3, A3 scallop of MV.

**FIGURE 4:**
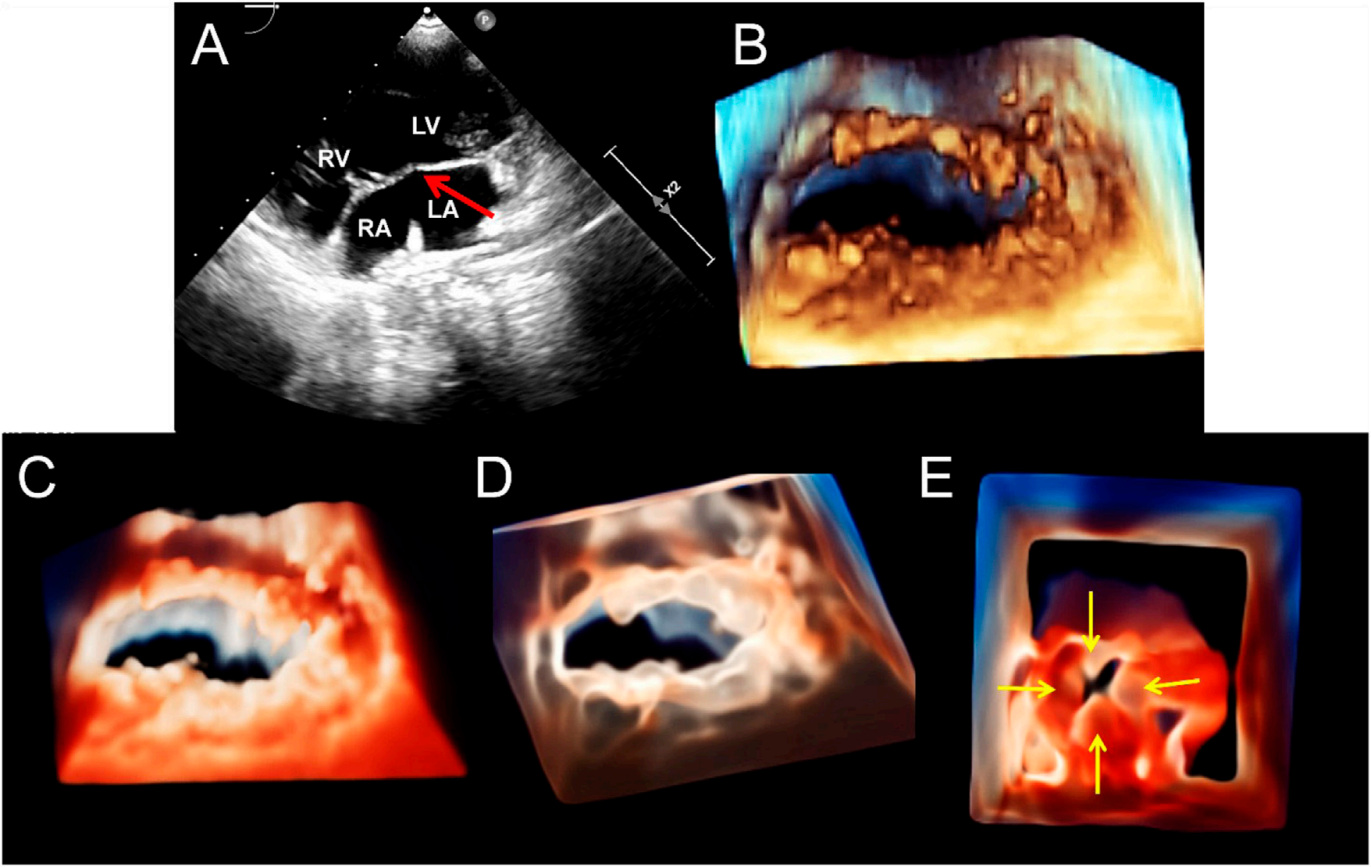
Transthoracic echocardiography shows complete atrioventricular septal defect. **(A)**. The four-chamber view of a 2D echocardiography shows only one atrioventricular valve (arrow), combined with a large defect of the lower part of the atrial septum and large ventricular septal defect. **(B–D)**. Traditional 3D, TrueVue, and TrueVue Glass show a single atrioventricular annulus and a group of atrioventricular valves open from the atrium view in diastole. **(E)**. TrueVue Glass shows the systolic phase with atrioventricular valve closed as a single annulus and four lobes from the perspective of the atrium, which are left, right, anterior, and posterior bridge lobes (arrows), confirmed to be a complete atrioventricular septal defect deformity. 2D, two-dimensional; 3D, three-dimensional. LA, left atrium; RA, right atrium; LV, left ventricle; RV, right ventricle.

**FIGURE 5:**
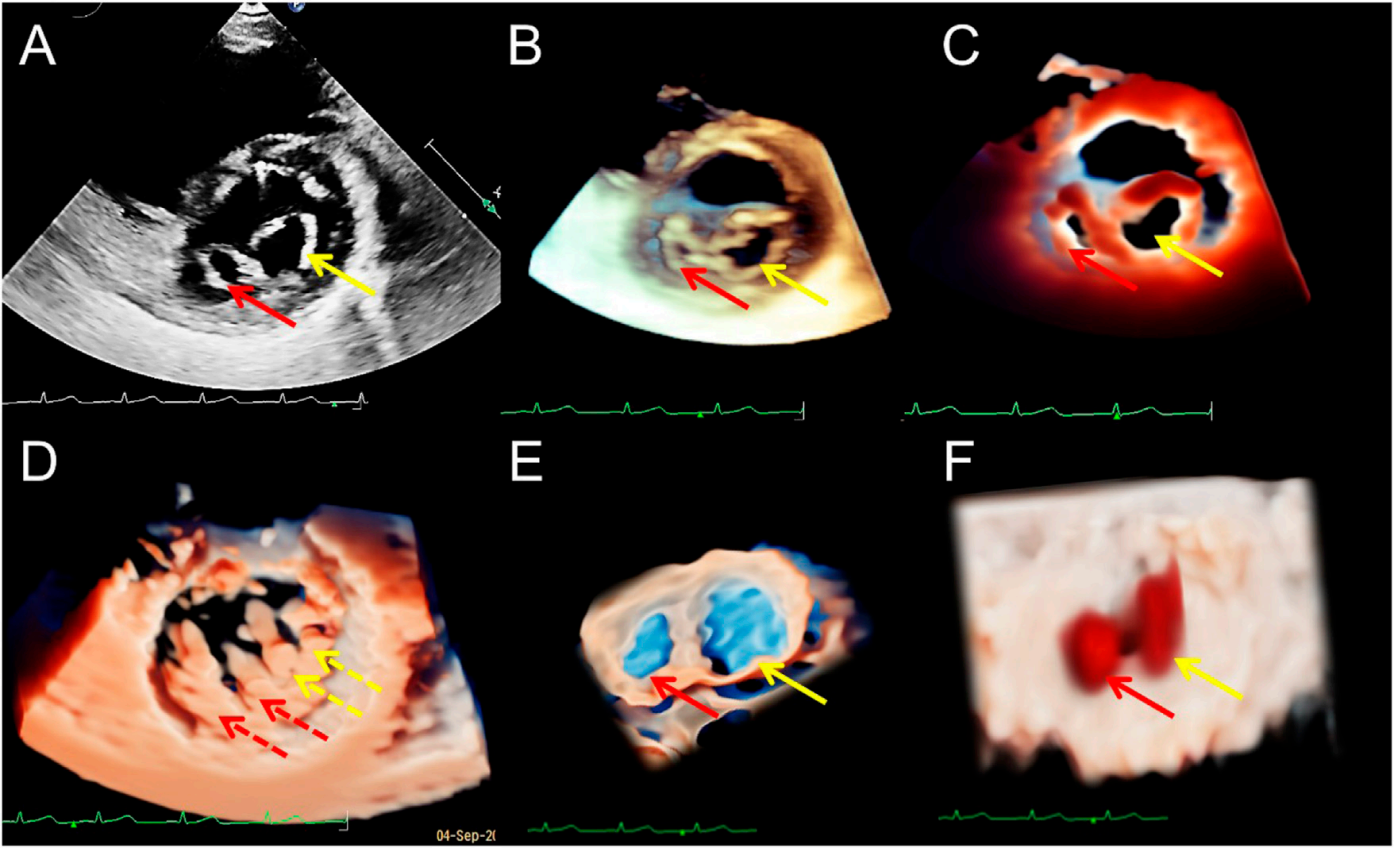
Transthoracic echocardiography of the short-axis view of the mitral valve (MV) showing the double orifice MV (arrows). **(A)**. 2D display of asymmetric orifices; **(B,C)**. Traditional 3D and TrueVue show the spatial shape of the orifices; **(D)**. TrueVue shows the abnormal multiple papillary muscles and the tendons connected to them from the perspective of the left ventricle; the red dotted arrows represent the additional structure corresponding to the small orifice, and the yellow dotted arrows represents the additional structure corresponding to the large orifice. TrueVue Glass **(E)** plus color Doppler **(F)** directly visualizes the two orifices of the MV from the left atrium side (surgical view) and the two blood streams entering the left ventricle during diastole. The red and yellow solid line arrows in **(A–C,E,F)** indicate two asymmetric orifices of MV, respectively. 2D, two-dimensional; 3D, three-dimensional.

**FIGURE 6:**
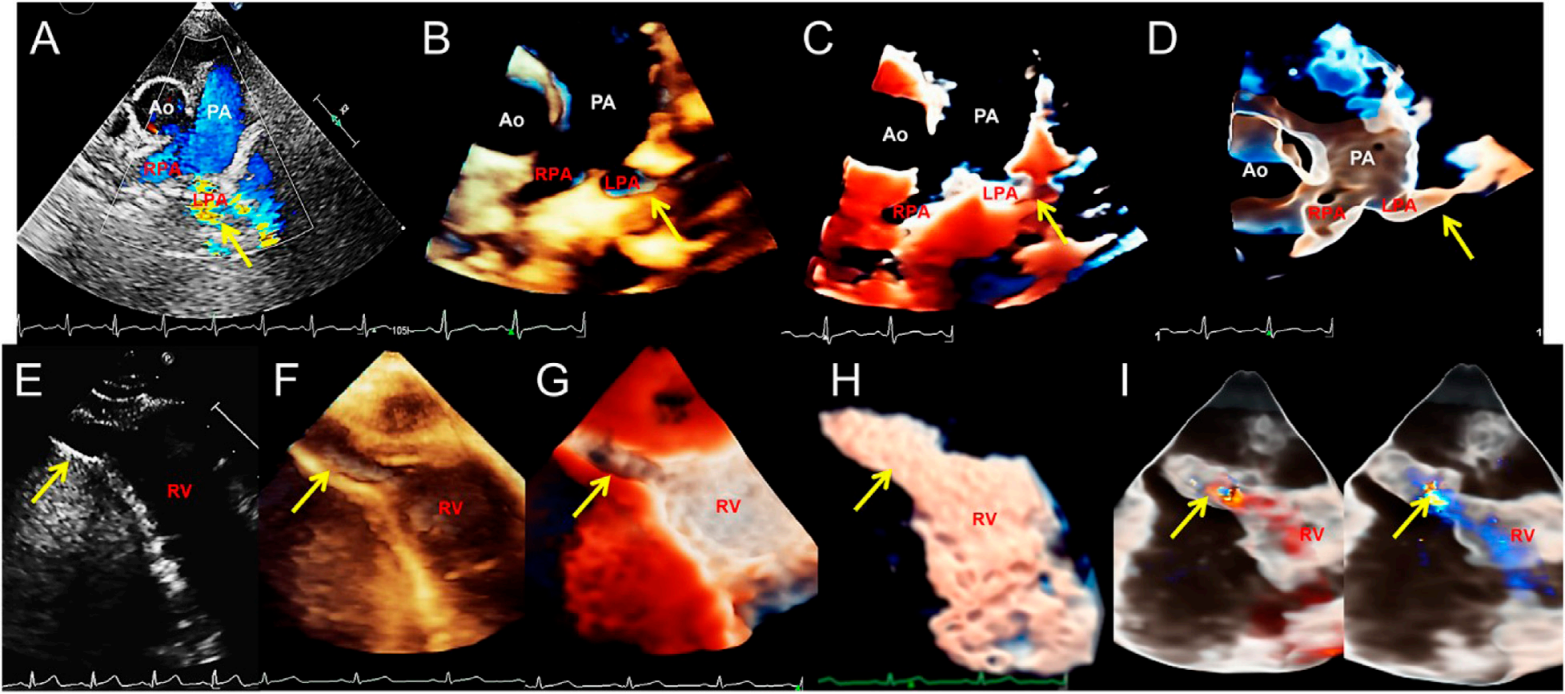
Transthoracic echocardiography shows pulmonary artery sling and right ventricular diverticulum. **(A–D)**. Transthoracic echocardiography showing the pulmonary artery sling on the short-axis section of the great artery. 2D, traditional 3D, TrueVue Light, and TrueVue Glass show abnormal orientation of the left pulmonary artery, which originate from a lower position, bypassing the rear of the trachea and then reflexed to the left, with local stenosis showing increased blood flow speed (arrows). **(E–H)**. 2D, traditional 3D, TrueVue Light, and TrueVue Glass show the diverticula (the end-systolic volume is 15 mm × 9 mm × 9 mm, and the end-diastolic volume is 24 mm × 14 mm × 14 mm, arrows) at the apex of the RV (arrows). **(I)**. TrueVue Glass with color Doppler shows that blood in the right ventricle enters the diverticulum during diastole (left), and blood flow in the diverticulum returns to the right ventricle during systole (right). 2D, two-dimensional; 3D, three-dimensional; Ao, aorta; PA, pulmonary artery; LPA, left pulmonary artery; RPA, right pulmonary artery; RV, right ventricle.

For patients undergoing innovative 3D imaging, 55 cases (88.7%) obtained satisfactory TrueVue images, and 59 (95.2%) obtained satisfactory TrueVue Glass images. For patients who underwent TTE, the overall satisfaction ratio with 3D images was 87.8% (36/41). For TEE, the overall satisfaction ratio was 100% (21/21). The standard for satisfactory image quality is to provide a clear and obvious evidence for diagnosis. Dissatisfaction with the quality of transthoracic ultrasound images is mainly due to obesity or excessive lung gas. During the TrueVue imaging of the patients with VSD, placing the light source below the defect made the shape and edge of the defect extremely eye-catching when compared with that of traditional 3D ultrasound, especially for the diagnosis of a small perimembranous ventricular defect (Figure 1). While in the patients with an ASD, placing the light source over the defect displayed the soft edge more clearly and accurately than traditional 3D imaging. After switching to the TrueVue Glass mode and appropriately increasing the transparency, the overall path and spatial range of the shunt through the defects was observed (Figure 2; Supplementary Movie S1). For the patients with PDA, TrueVue Glass plus color Doppler technology better displayed the origin, course, and inner diameter of the shunt of the whole heart cycle in the pulmonary artery through the transparent tube wall structure than traditional 3D or TrueVue imaging modes (Supplementary Movie S2), especially in the synchronous double-sided view (dual volume mode) (Supplementary Figure S1). For the patients with bicuspid aortic valve (BAV) malformation, the valve displayed by TrueVue more closely simulated the real anatomical texture features and showed the crest-like raised fusion structure between the two valves (Supplementary Figure S2). Although both 2D and 3D imaging modes can diagnose BAV, the new imaging mode displays images that are closer to real anatomy and will make communication with surgeons and patients easier and more efficient. For complex mitral clefts, observation of the position, number, shape, and size of the clefts was significantly improved on the TrueVue Light and Glass compared with traditional technologies; furthermore, Glass imaging was more advantageous for irregularly shaped clefts boundaries (Figure 3). In patients with pulmonary artery stenosis, 2D, traditional 3DE, and TrueVue showed echo enhancement, restricted opening of the pulmonary valve, and the turbulence signal when blood passed through the stenotic position. However, in the TrueVue Glass mode, the open state of the flap was clearly displayed according to the light transmittance, and because of the transparence of the surrounding tissue, a segment of the right ventricular outflow tract or the main pulmonary artery was revealed, enabling identification of the location of the opening margin for the pulmonary valve more easily and accurately (Supplementary Figure S3). When CAVSD were suspected on 2DE, TrueVue Light made it easier to determine the number of annulus, while TrueVue Glass showed several bridge lobes of the common atrioventricular valve (Figure 4). For patients with a rare congenital DOMV, novel 3D images not only illustrated the two-orifice structure, but also showed the thickness and arrangement of the chordae and papillary muscles (Figure 5). Pulmonary artery sling is difficult to be observed on 2D and traditional 3DE image, and there was little improvement over traditional 3DE when converting to TrueVue Light, TrueVue Glass displayed an unprecedented visual perspective of the abnormal left pulmonary artery (Figure 6). Finally, in the patient with rare ventricular diverticulum, placing the light source of TrueVue at the entrance of the diverticulum significantly improved the clarity of the patient’s finger-shaped diverticulum through the shadow effect when compared with the image of traditional 3DE. Meanwhile, TrueVue Glass shielded the structure around the diverticulum, so that only the shape of the diverticulum was directly indicated (Figure 6), with the addition of color Doppler, the blood flow in and out of the diverticulum with the cardiac cycle clearly appears (Supplementary Movie S3).

### Clinical rating and scoring survey results

Survey Data results showed that the novel 3D imaging methods effectively increased the diagnostic confidence of echocardiographers, enabled surgeons and patients to better understand the details of lesions, promoted the efficiency of communication, and improved the confidence of both doctors and patients in treatment (*p* < 0.0001) (Table 1).

## Discussion

TrueVue is a new, high definition 3D rendering mode whose spatial image gradually transitions from light-pink to orange-red to represent the surface to deep structures and simulate the real texture of heart tissue, enabling a hierarchical and realistic view of a variety of structures (Genovese et al., 2019; Kern et al., 2019; Vainrib et al., 2019; Vairo et al., 2019; Merino Argos et al., 2020). On the other hand, the recent novel TrueVue Glass intelligent hides the myocardium and tissue surrounding the heart that contains little blood automatically, transforming the image into a translucent mode to achieve a clear spatial relationship between the internal structure and the geometry of the heart chamber and vessels structure, information that has never been provided by conventional cardiac ultrasonic images after birth (Karagodin et al., 2020). TrueVue’s raw stereo data acquisition and cutting methods are similar to those of traditional 3D, no additional operational steps are added. With TrueVue Light, that is, using a touch screen to retract and/or rotate the TrueVue or TrueVue Glass image and adding a movable virtual light source (Genovese et al., 2019), the area of interest can be illuminated or darkened by adjusting the observation angle and the position and depth of the light source to improve the display ability and detail effects, which enables doctors to have a clearer understanding of the overall anatomical structure of the heart.

Although traditional 3DE can display some congenital malformations, there remain shortcomings in the resolution of small structures and the display of deeper organizational structures (Ge, 2010; Khoshhal, 2013; Charakida et al., 2014; Cossor et al., 2015). Therefore, the preoperative evaluation and intraoperative guidance of CHD may require other relatively expensive and time-consuming imaging techniques (Silvestry et al., 2014; Jone et al., 2016; Isorni et al., 2020). The novel TrueVue, TrueVue Light, and TrueVue Glass series of 3DE overcome these deficiencies to some extent, providing more detailed and accurate information for CHD both in the preoperative diagnosis and postoperative follow-up.

Although the diagnostic value of traditional 3DE has been confirmed for relatively common septal defects (Saric et al., 2010; Charakida et al., 2014; Cossor et al., 2015; Hadeed et al., 2016), TrueVue plus a light source makes not only the defect boundary clearer, but also makes the range for the soft rim more visible. In TrueVue Glass, adjusting the transparency highlights the origin and extent of an atrial or ventricular shunt. For PDAs, TrueVue Glass with color allows us to observe the pattern of abnormal blood flow, especially when using the “dual” mode, where we can see that the shunt bundle in the deep pulmonary artery may be more extensive than the surface flow seen in the 2D view. These images can help us judge the PDA typing and degree, because even if the opening width is the same, the shunt of the “window-type” may be much greater than that of the long “tube-type” (Youssef et al., 2020; Sehgal et al., 2021).

For malformations of the thin cardiac valves, traditional 3D images are closely dependent on the quality of 2D images; however, while they are often used to evaluate the relatively large and morphologically regular mitral valves (Sun et al., 2017; Kalçık et al., 2021), their diagnostic value for pulmonary valves is considered to be very limited and thus have not been routinely used (Anwar et al., 2007; Valente et al., 2014). TrueVue Light imaging is advantageous for the mitral and aortic valves. Moreover, although the image quality is improved for the pulmonary valve, the display is still restricted when the quality of the original 2D image is poor, which is not enough to provide sonographers with sufficient diagnostic confidence, especially for infants or for TTE screening. Under these circumstances, the application of TrueVue Glass may be helpful, as its display of the location, number, width, and overall morphology of the mitral valve multiple clefts and pulmonary stenosis is significantly improved, and it provides a clear surgical field of view. In endocardial cushion malformation, it is often difficult to distinguish between “transitional” or complete “single common annulus” on 2D ultrasound (Nina et al., 2019). TrueVue Light imaging makes it quite easy to determine the number of annulus and the morphology of the atrioventricular valve. The shape and outline of the thin bridge leaflet in CAVSD can be clearly displayed when TrueVue Glass is also applied. DOMV is often accompanied by multiple sets of papillary muscle malformations (Karas et al., 2003; Erkol et al., 2009). Traditional 2D and 3D ultrasound can exactly diagnose the existence of the double orifice, but it is often difficult to display the additional structure of the mitral valve apparatus. The TrueVue Light more clearly illuminates the structural characteristics connecting the chordae tendineae to the corresponding papillary muscles.

This is exactly the issue with 3D rendering techniques: they often “look good” but misrepresent the anatomic details; especially with thin structures. The new Glass imaging mode has obvious visual enhancement effects on the ability to display thin structures, such as the atrial septum and thin valves, to a certain extent, improving the problem of false echo loss in traditional ultrasound.

Rare pulmonary artery sling imaging has certain difficulties in 2D and traditional 3DE, usually need time-consuming and laborious to confirm the diagnosis through the 3D reconstruction of enhanced computed tomography (CT), and the children under examination are not easy to cooperate well (Sezer et al., 2019; Xu et al., 2020). TrueVue Glass has an obvious advantage in the diagnosis of pulmonary artery sling, and it is the first time that an innovative 3D ultrasound technology has been used to diagnose such rare cardiovascular malformations. Rare ventricular diverticulum, with its narrow entrance and long shape, may be easily missed or misdiagnosed as a paracardiac vessel on 2D ultrasound (Sozzi et al., 2017; Wang et al., 2021). The TrueVue Light highlights the interior space features of the diverticulum. TrueVue Glass further presents the outer contour of the diverticulum directly. Previously, this contour could only be seen with 3D reconstruction of enhanced CT.

Because the content and features of the diagnostic concerns of different diseases are significantly different when applying ultrasound imaging, we will ask more targeted questions for different diseases during the questionnaire process, also because the diagnostic advantages of these new 3D imaging technologies will be more obvious for certain types of CHD. At the same time, considering that the patient’s feelings are equally important in the evaluation and treatment of CHD, including a full understanding of the characteristics and extent of their disease and the surgical procedure explained by the doctor, we ask some of the patients or their guardians to observe the conventional and new 3D display of the lesion through the doctor’s explanation of the characteristics of their disease. However, since they are not professionally trained in medicine, they will only answer a small part of the questions related to their medical procedure.

Although the true resolution of the above-mentioned new series of 3D images has not been improved digitally, these new imaging modes plus light and shadow effects have significantly improved the visual effects and diagnostic efficiency of the characteristics of CHDs. Although traditional 2D and 3DE can also help us complete most of the ultrasonic diagnosis of CHD, it is time-consuming and labor-intensive, especially in some special diseases. The innovative TrueVue combined with Light and Glass 3D imaging technology provides us with more reliable reference information. In particular, the layered sense by the photorealistic light source addition, and the outer contour imaging of the heart cavity displayed by the application of Glass (such as the shape of the left atrial appendage (Karagodin et al., 2020) and the rare cardiac diverticulum, etc.) are new image forms and diagnostic information that have never been obtained by traditional postpartum cardiac ultrasound diagnostic technology, which improve and correct the diagnosis more efficiently.

### Limitations

The TrueVue, TrueVue Light, and TrueVue Glass series of 3D cardiac ultrasound technologies have achieved satisfactory results in some research fields, but there are some limitations and deficiencies. This innovative series of 3DE improved the visual clarity of image details to a certain extent, but in cases where the 2D image was very dissatisfied displayed, it was still difficult to obtain a satisfying 3D image, especially in TrueVue mode. Moreover, the application of TrueVue plus light and Glass have requirements for the operator, who should have mastery of the overall and partial anatomy of the heart and be able to apply the tools of the machine for image processing. It also requires the operator to be an “artist,” able to flexibly use the visual effects produced by the light source to display the abnormal anatomical structure. Therefore, a certain learning curve to obtain optimal rendered images is required, and for doctors with traditional 3DE operation experience, it is easy to master the methods and skills of the new series of 3DE operations. Furthermore, the extensive post-processing increases the possibility of artificial creation or loss of defects based on variations in both scan quality and post-processing technique. At present, as the 3DE of the TrueVue series, especially TrueVue Glass, is a recently launched novel tools, there are few published reports about them. In addition, although our research is one with the largest sample size reporting on the application of the above-mentioned new tools, it discusses limited types of CHDs. Therefore, further studies are needed to explore their application value and experience.

## Conclusion

This study showed the excellent applicability of TrueVue, TrueVue Light, and TrueVue Glass as diagnostic tools for patients with suspected CHD. Compared with 2D and traditional 3DE, the TrueVue, TrueVue Light, TrueVue Glass series of novel 3DE technologies’ display of abnormal structures in patients with CHD more closely simulate real anatomical features and make the outer contours of the heart chambers, the thin valves and blood flow in the lumen more comprehensive and clearer. These technologies provide us with a wealth of evidence for the diagnosis and treatment of CHD and are a revolution in the ultrasonic diagnosis method of CHD.

## Data Availability

The original contributions presented in the study are included in the article/Supplementary Material, further inquiries can be directed to the corresponding author.

## Supplementary material

SUPPLEMENTARY FIGURE S1 The diagnosis of patent ductus arteriosus by transthoracic echocardiographic short-axis section of the great artery. . 2D, traditional 3D and TrueVue Light plus color Doppler showing the shunt from the aorta to the main pulmonary artery (3 mm, arrows). **(D)**. TrueVue Glass “dual volume” shows the ventral and dorsal flow of the pulmonary artery, with shunt signals from the ducts more dorsal in space (arrows). 2D, two-dimensional; 3D, three-dimensional.

SUPPLEMENTARY FIGURE S2 Transesophageal echocardiography showed bicuspid aortic valve malformation. . 2D, traditional 3D, TrueVue Light, and TrueVue Glass show the fusion crest by the left coronary and non-coronary leaflet (arrows) from the left ventricular outflow tract side perspective, respectively. 2D, two-dimensional; 3D, three-dimensional.

SUPPLEMENTARY FIGURE S3 . 2D transthoracic echocardiographic short-axis view of the great artery demonstrating a marked acceleration of blood flow (arrow) through the pulmonary valve. **(B)**. Conventional 3D imaging from the right ventricular outflow tract demonstrates a trilobed pulmonary valve with thickened fusion at the valve tip (arrow). **(C)**. TrueVue shows the thickened tip of the pulmonary valve (arrow), but the thin lobes show pseudo-echogenic loss. **(D)**. TrueVue Glass shows thin valves and inadequately opening valves (arrow) during systole. Figures **(B-D)** are from the perspective of the right ventricular outflow tract. 2D, two-dimensional; 3D, three-dimensional.

SUPPLEMENTARY MOVIE S1 TrueVue Glass plus color Doppler shows left atrial blood flow through the atrial septal defect (arrow) into the right atrium on two-atrial section of transesophageal echocardiography. LA, left atrium; RA, right atrium.

SUPPLEMENTARY MOVIE S2 The TrueVue Glass plus color Doppler shows the flow from the aortic arch into the pulmonary artery through an open ductal artery (arrow) on supra sternal fossa section by thoracic echocardiography. AoA, aortic arch; PA, pulmonary artery.

SUPPLEMENTARY MOVIE S3 The TrueVue Glass plus color Doppler shows blood shuttling between the right ventricle and diverticulum (arrow) during the systolic and diastolic phases of the heart. RV, right ventricle.

## Abbreviations

2DE, Two-dimension echocardiography; 3DE, Three-dimensional echocardiography; AI, Artificial intelligence; ASD, Atrial septal defect; AV, Aortic valve; CHD, Congenital heart disease; DOMV, Double orifice mitral valve; CAVSD, Complete atrioventricular septal defect; MV, Mitral valve; PDA, Patent ductus arteriosus; PS, Pulmonary artery stenosis; TEE, Transesophageal echocardiography; TTE, Transthoracic echocardiography; VSD, Ventricular septal defect.

## References

[B1] Anwar A. M. Soliman O. Bosch A. E. V. D. Mcghie J. S. Meijboom F. J. ten Cate F. J. (2007). Assessment of pulmonary valve and right ventricular outflow tract with real-time three-dimensional echocardiography. Int. J. Cardiovasc. Imaging 23, 167–175. 10.1007/s10554-006-9142-3 16960754

[B2] Charakida M. Pushparajah K. Anderson D. Simpson J. M. (2014). Insights gained from three-dimensional imaging modalities for closure of ventricular septal defects. Circ. Cardiovasc. Imaging 7, 954–961. 10.1161/CIRCIMAGING.114.002502 25406198

[B3] Cossor W. Cui V. W. Roberson D. A. (2015). Three-dimensional echocardiographic en face views of ventricular septal defects: Feasibility, accuracy, imaging protocols and reference image collection. J. Am. Soc. Echocardiogr. 28, 1020–1029. 10.1016/j.echo.2015.05.014 26141981

[B4] Erkol A. Karagoz A. Ozkan A. Koca F. Yilmaz F. Sonmez K. (2009). Double-orifice mitral valve associated with bicuspid aortic valve: A rare case of incomplete form of shone's complex. Eur. J. Echocardiogr. 10, 801–803. 10.1093/ejechocard/jep067 19502622

[B5] Fabricius A. Walther T. Falk V. Mohr F. (2004). Three-dimensional echocardiography for planning of mitral valve surgery: Current applicability? Ann. Thorac. Surg. 78, 575–578. 10.1016/j.athoracsur.2003.10.031 15276524

[B6] Ge S. (2010). How can we best image congenital heart defects? Are two-dimensional and three-dimensional echocardiography competitive or complementary? J. Am. Soc. Echocardiogr. 23, 722–725. 10.1016/j.echo.2010.05.018 20620860

[B7] Genovese D. Addetia K. Kebed K. Kruse E. Yamat M. Narang A. (2019). First clinical experience with 3-dimensional echocardiographic transillumination rendering. JACC. Cardiovasc. Imaging 12, 1868–1871. 10.1016/j.jcmg.2018.12.012 30772235 PMC7538076

[B8] Hadeed K. Hascoet S. Amadieu R. Karsenty C. Cuttone F. Leobon B. (2016). Assessment of ventricular septal defect size and morphology by three-dimensional transthoracic echocardiography. J. Am. Soc. Echocardiogr. 29, 777–785. 10.1016/j.echo.2016.04.012 27289424

[B9] Harake D. Gnanappa G. K. Alvarez S. G. V. Whittle A. Punithakumar K. Boechler P. (2020). Stereoscopic display is superior to conventional display for three-dimensional echocardiography of congenital heart anatomy. J. Am. Soc. Echocardiogr. 33, 1297–1305. 10.1016/j.echo.2020.06.016 32919855

[B10] Isorni M. A. Moisson L. Moussa N. B. Monnot S. Raimondi F. Roussin R. (2020). 4D flow cardiac magnetic resonance in children and adults with congenital heart disease: Clinical experience in a high volume center. Int. J. Cardiol. 320, 168–177. 10.1016/j.ijcard.2020.07.021 32712110

[B11] Jone P. N. Ross M. M. Bracken J. A. Mulvahill M. J. Di Maria M. V. Fagan T. E. (2016). Feasibility and safety of using a fused echocardiography/fluoroscopy imaging system in patients with congenital heart disease. J. Am. Soc. Echocardiogr. 29, 513–521. 10.1016/j.echo.2016.03.014 27143284

[B12] Kalçık M. Özkan M. Gündüz S. Gürsoy M. Yesin M. Bayam E. (2021). Normal reference values for mechanical mitral prosthetic valve inner diameters and areas assessed by two-dimensional and real-time three-dimensional transesophageal echocardiography. Int. J. Cardiovasc. Imaging 37, 547–557. 10.1007/s10554-020-02039-5 33011903

[B13] Karagodin I. Addetia K. Singh A. Dow A. Rivera L. DeCara J. M. (2020). Improved delineation of cardiac pathology using a novel three-dimensional echocardiographic tissue transparency tool. J. Am. Soc. Echocardiogr. 33, 1316–1323. 10.1016/j.echo.2020.08.005 32972777 PMC7920620

[B14] Karas S. Barbetseas J. Lambrou S. Parissis J. Metzikof D. Toutouzas P. (2003). Well-functioning double-orifice mitral valve in a young adult. J. Clin. Ultrasound. 31, 170–173. 10.1002/jcu.10142 12594805

[B15] Kern M. C. Janardhanan R. Kelly T. Fox K. A. Klewer S. E. Seckeler M. D. (2019). Multimodality imaging for diagnosis and procedural planning for a ruptured sinus of Valsalva aneurysm. J. Cardiovasc. Comput. Tomogr. 14, e139–e142. 10.1016/j.jcct.2019.09.018 31587967

[B16] Khoshhal S. (2013). Feasibility and effectiveness of three-dimensional echocardiography in diagnosing congenital heart diseases. Pediatr. Cardiol. 34, 1525–1531. 10.1007/s00246-013-0718-0 23677391

[B17] Meras P. Riesgo-Gil F. Rybicka J. Barradas-Pires A. Smith J. Kempny A. (2021). Heart transplantation at a single tertiary adult congenital heart disease centre: Too little, too late? Int. J. Cardiol. 322, 107–113. 10.1016/j.ijcard.2020.08.047 32798622

[B18] Merino Argos C. Lopez Fernandez T. Valbuena Lopez C. (2020). Severe stenosis due to prosthetic thrombosis evaluated with TrueVue. Rev. Esp. Cardiol. 73, 499. 10.1016/j.rec.2019.07.015 31631047

[B19] Nina H. Thomas H. Torsten M. Wierup P. Maynard C. Ramgren Johansson J. (2019). Comprehensive echocardiographic imaging of atrioventricular valves in children with atrioventricular septal defect: Accuracy of 2D and 3D imaging and reasons for disagreement. Anatol. J. Cardiol. 21, 214–221. 10.14744/AnatolJCardiol.2019.49376 30930449 PMC6528495

[B20] Saric M. Perk G. Purgess J. R. Kronzon I. (2010). Imaging atrial septal defects by real-time three-dimensional transesophageal echocardiography: Step-by-step approach. J. Am. Soc. Echocardiogr. 23, 1128–1135. 10.1016/j.echo.2010.08.008 20833505

[B21] Sehgal A. Nitzan I. Krishnamurthy M. Pharande P. Tan K. (2021). Toward rational management of patent ductus arteriosus: Ductal disease staging and first line paracetamol. J. Matern-Fetal Neo Med. 34 (23), 3940–3945. 10.1080/14767058.2019.1702949 31885289

[B22] Sezer S. Acar D. Ekiz A. Kaya B. Bornaun H. Aslan H. (2019). Prenatal diagnosis of left pulmonary artery sling and review of literature. Echocardiography 36, 1001–1004. 10.1111/echo.14325 30968436

[B23] Silvestry F. E. Kadakia M. B. Willhide J. Herrmann H. C. (2014). Initial experience with a novel real-time three-dimensional intracardiac ultrasound system to guide percutaneous cardiac structural interventions: A phase 1 feasibility study of volume intracardiac echocardiography in the assessment of patients with structural heart disease undergoing percutaneous transcatheter therapy. J. Am. Soc. Echocardiogr. 27, 978–983. 10.1016/j.echo.2014.04.022 24930123

[B24] Simpson J. Lopez L. Acar P. Friedberg M. Khoo N. Ko H. (2017). Three-dimensional echocardiography in congenital heart disease: An expert consensus document from the European association of cardiovascular imaging and the American society of echocardiography. J. Am. Soc. Echocardiogr. 30, 1–27. 27838227 10.1016/j.echo.2016.08.022

[B25] Sozzi F. Hugues N. Civaia F. Alexandrescu C. Iacuzio L. (2017). Multimodality imaging of a congenital diverticulum of the left ventricular outflow tract. Eur. Heart J. Cardiovasc. Imaging 18, 144. 10.1093/ehjci/jew170 27550665

[B26] Sun F. Chen Y. Ren W. Zhang Y. Wu D. Chen X. (2017). Four-tiered echocardiographic analysis approach for congenital mitral valve malformations: Four years of experience. Int. J. Cardiol. 227, 602–610. 10.1016/j.ijcard.2016.10.090 27814918

[B27] Vainrib A. F. Bamira D. Aizer A. Chinitz L. A. Loulmet D. Benenstein R. J. (2019). Photorealistic imaging of left atrial appendage occlusion/exclusion. Echocardiography 36, 1601–1604. 10.1111/echo.14438 31385344

[B28] Vairo A. Marro M. De Ferrari G. M. Rinaldi M. Salizzoni S. (2019). Use of a photo-realism 3D rendering technique to enhance echocardiographic visualization of the anatomical details during beating-heart mitral valve repair. Echocardiography 36, 2090–2093. 10.1111/echo.14515 31682031

[B29] Valente A. Cook S. Festa P. Ko H. Krishnamurthy R. Taylor A. (2014). Multimodality imaging guidelines for patients with repaired tetralogy of fallot: A report from the AmericanSsociety of echocardiography: Developed in collaboration with the society for cardiovascular magnetic resonance and the society for pediatric radiology. J. Am. Soc. Echocardiogr. 27, 111–141. 10.1016/j.echo.2013.11.009 24468055

[B30] van den Bosch A. Ten Harkel D. McGhie J. Roos-Hesselink J. Simoons M. Bogers A. (2006). Surgical validation of real-time transthoracic 3D echocardiographic assessment of atrioventricular septal defects. Int. J. Cardiol. 112, 213–218. 10.1016/j.ijcard.2005.09.012 16303189

[B31] Wang Y. Liu C. Yin A. Zhao X. He W. Xiong Y. (2021). Prenatal diagnosis of fetal right ventricular diverticulum with massive pericardial effusion in one of monochorionic diamniotic twins: A case report with a favorable outcome following *in utero* pericardiocentesis. J. Int. Med. Res. 49, 300060520986668. 10.1177/0300060520986668 33478307 PMC7841867

[B32] Xu L. Wang L. Gai Y. Ma Q. Xiang G. Ouyang C. (2020). Prenatal sonographic diagnosis of partial left pulmonary artery sling: A rare case report. J. Clin. Ultrasound. 49, 257–261. 10.1002/jcu.22913 32929784

[B33] Youssef D. Flores M. Ebrahim E. Eshak K. Westerink J. Chaudhuri D. (2020). Assessing the clinical significance of echocardiograms in determining treatment of patent ductus arteriosus in neonates. J. Neonatal. Perinat. Med. 13, 345–350. 10.3233/NPM-170122 32925117

