# Supplementary figures and images for: Novel TrueVue series of 3D echocardiography: Revealing the pathological morphology of congenital heart disease
Source: Front Physiol. 2022 Sep 6;13:1000007. doi: 10.3389/fphys.2022.1000007 (PMC9485809; doi:10.3389/fphys.2022.1000007)

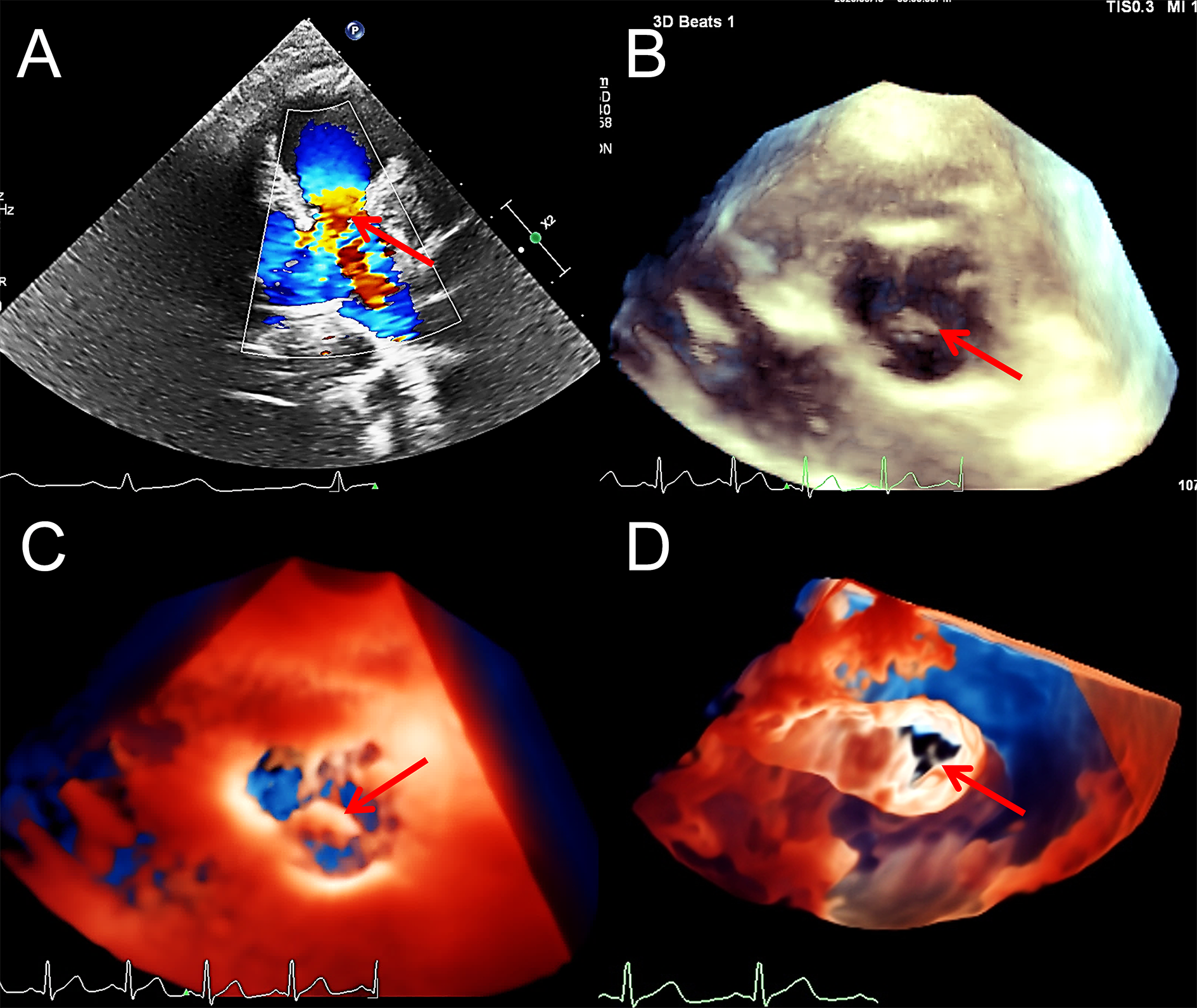

Supplement: Supplementary file 2 [file Image3.TIF]

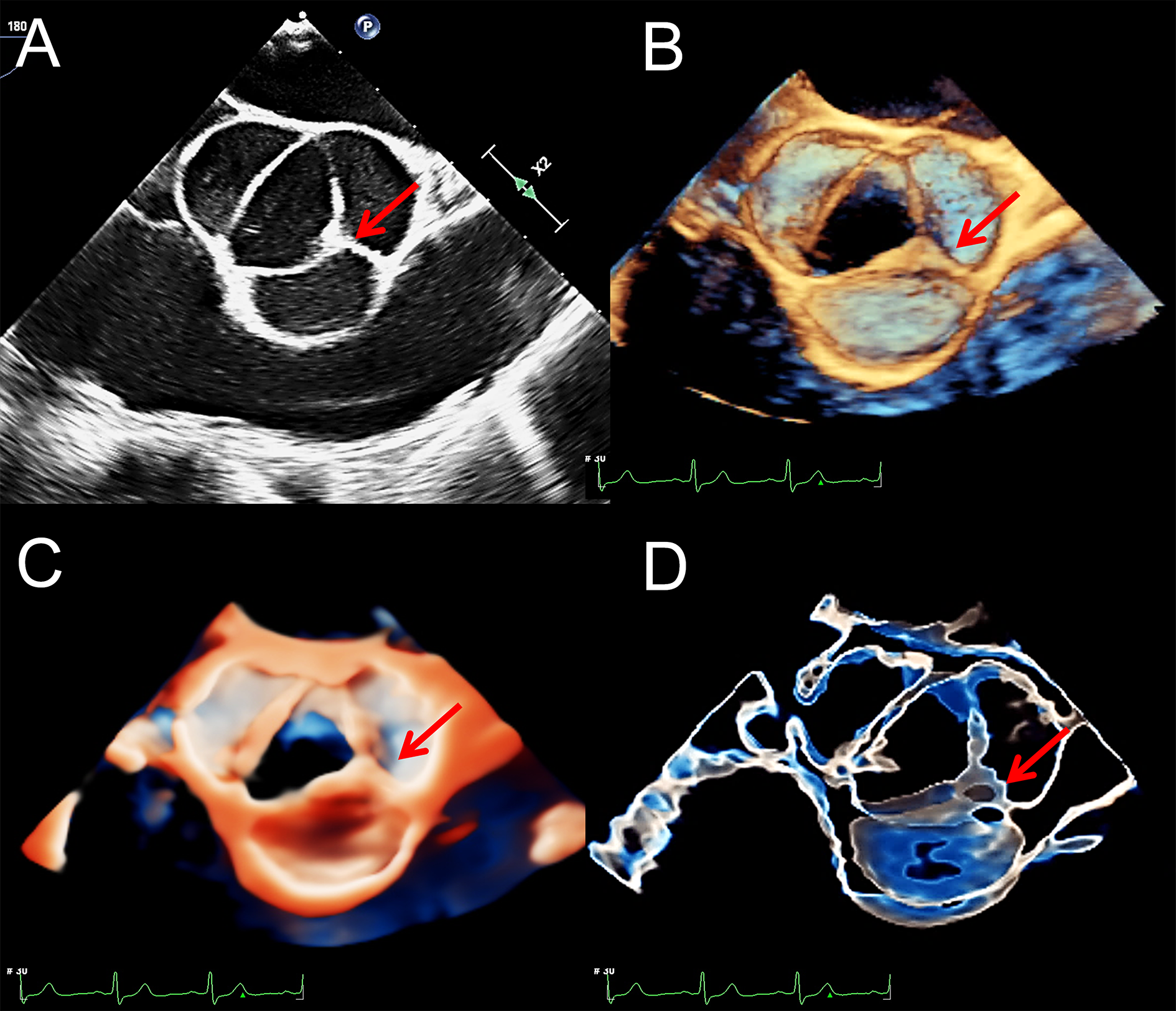

Supplement: Supplementary file 3 [file Image2.TIF]

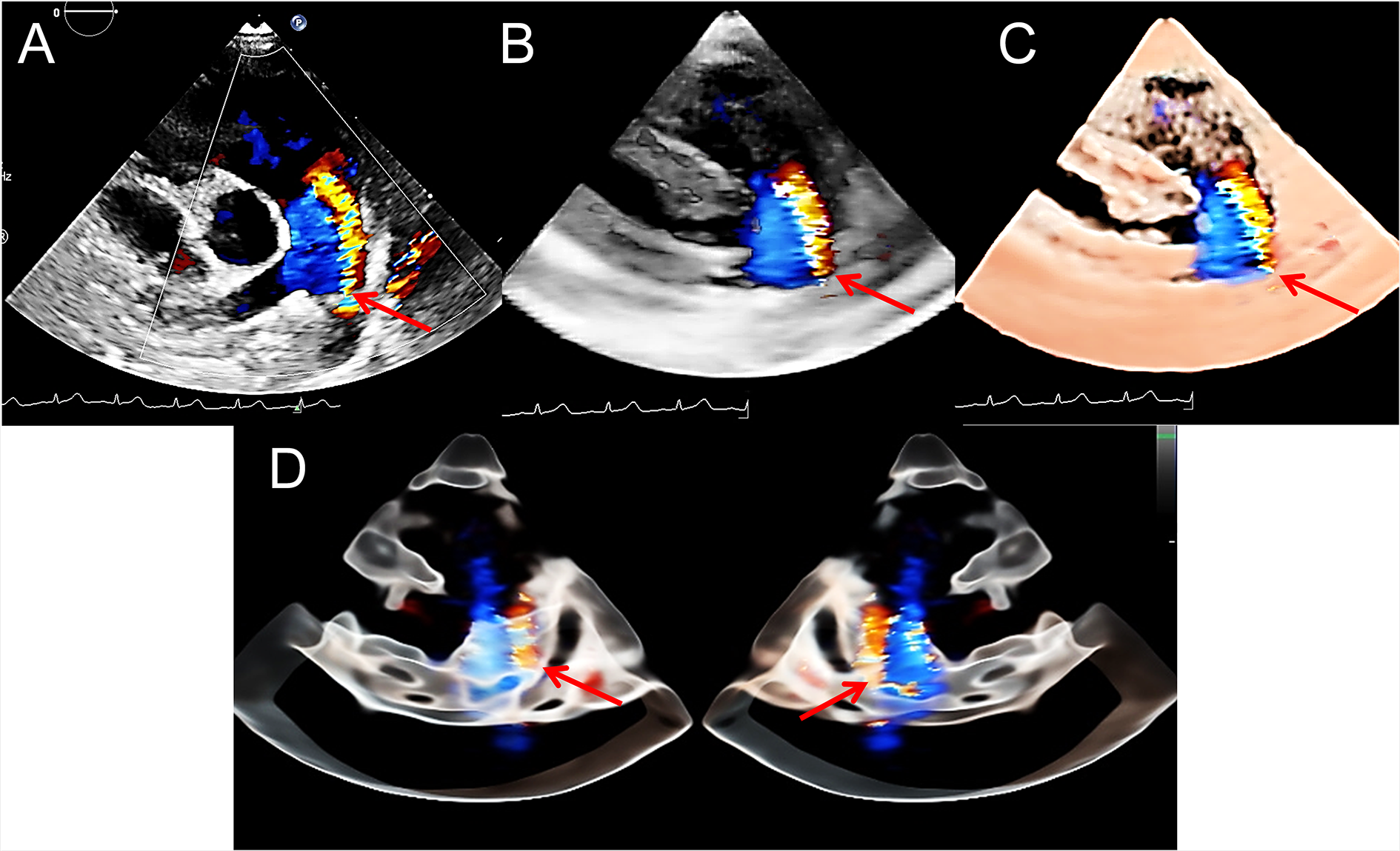

Supplement: Supplementary file 4 [file Image1.TIF]
